# Bridging healthcare access: strategies beyond the COVID-19 public health emergency

**DOI:** 10.1007/s44250-024-00100-x

**Published:** 2024-08-20

**Authors:** Amaya Najma Razmi, Simar S. Bajaj, Fatima Cody Stanford

**Affiliations:** 1 Harvard University, Cambridge, MA USA; 2 Department of Medicine-Division of Endocrinology-Neuroendocrine, Massachusetts General Hospital, MGH Weight Center, 50 Stanford Street, 4th Floor, Boston, MA USA; 3 Department of Pediatrics-Division of Endocrinology, Nutrition Obesity Research Center at Harvard (NORCH), Harvard Medical School, Boston, MA USA

**Keywords:** health equity, COVID-19, public health

## Abstract

Issued in January 2020, the federal Public Health Emergency (PHE)’s termination was ultimately inevitable and has prompted reflection over how the pandemic elicited relatively progressive reforms to healthcare. Although we are concerned that the PHE’s termination poses a significant threat to public health and equity, we believe that physicians, along with systemic changes, can provide critical support for patients as they navigate a shifting health policy landscape. In response to this evolving landscape, the article emphasizes the pivotal role of physicians and healthcare institutions in safeguarding patient access to care. It proposes strategies such as community-based workshops, patient navigators, and streamlined technology-driven redetermination processes to support vulnerable populations during this transition. Physicians are encouraged to engage in advocacy efforts, from voicing concerns at health meetings to collaborating with non-profit organizations and the media, to influence data-driven policy changes that prioritize patient safety and equitable access. Marginalized patients should not be slipping through the cracks.

Uncertainty wrought by the COVID-19 pandemic triggered a series of waivers and flexible health policies to ensure continued access to care. Issued in January 2020, the federal Public Health Emergency (PHE) enabled, among other policies, telehealth expansion, continuous Medicaid enrollment, and regulatory flexibilities for medications for opioid use disorder (MOUD). However, in May 2023, President Biden announced the end of the PHE. While this termination was ultimately inevitable, it has prompted reflection over how the pandemic elicited relatively progressive reforms to healthcare. Although we are concerned that the PHE’s termination poses a significant threat to public health and equity, we believe that physicians, along with policymakers, can provide critical support for patients as they navigate a shifting health policy landscape.

Already, the PHE’s termination has wrought a motley of stopgap measures to bridge access to care. In particular, the Consolidated Appropriations Act of 2023 extended some Medicaid and Medicare telehealth and substance use disorder flexibilities for another two years. These flexibilities include allowing the patient’s home as an originating site for telehealth services, extending reimbursement for audio-only services, and providing federally qualified health centers and rural health clinics the ability to provide telehealth services [1]. Over the course of the PHE, individuals in low-income groups experienced the greatest increase in telehealth uptake compared to higher income groups [2]. Furthermore, the Substance Abuse and Mental Health Services Administration has expanded methadone take-home dose access through 2024, an intervention that decreased emergency department visits due to overdoses from 40.4% pre-COVID to 30.6% in 2021 [3, 4]. Separately, the Biden Administration announced the temporary “Bridge Access Program” will continue to provide COVID-19 vaccines at no cost to uninsured individuals [5]. Although these measures are an important step toward preserving regulations, they are only band-aid solutions, with gaps still existing in the regulations.

In particular, the requirement of continuous eligibility for Medicaid patients has been dropped without remedy. Correspondingly, nearly 92.3 million Medicaid enrollees will have their benefits reevaluated over the next 9 to 12 months [6]. In Idaho, for instance, nearly 76% of individuals whose eligibility was checked were terminated, and nearly 2 million patients nationally have been terminated as of July 2023 [7], with the leading reasons for losing coverage being moving to a new state or having a household income that is too high to qualify via the modified adjusted gross income methodology [8].

Medicaid redetermination also exacerbates racial and ethnic inequities; models predict that the patients most impacted are underrepresented minorities and non-native English speakers who may not have the resources to properly file for redetermination or apply for coverage through the Affordable Care Act Marketplace. Other patients could end up falling between the cracks with incomes that are too low to qualify for subsidized insurance through the marketplace but too high to qualify for Medicaid. Without insurance, individuals are over twice as likely to forego medical care due to costs and, if they do receive medical care, spend much of their income on medical expenses and subsequently face financial difficulty [9].

In addition to systemic efforts to protect Medicaid access, hospitals and clinics can dedicate resources to support at-risk enrollees during redetermination so that patients can access the care they need. Physicians are uniquely positioned for this task since they are often a touch point for many underserved communities; not only are they the primary provider of services such as preventative care or chronic disease management, but they also often participate in community health centers or free clinics for vulnerable individuals. As a result, physicians often have already built trust and rapport with patients over time such that they are able to provide them with adequate information to navigate a complex and constantly shifting health policy landscape. Clinics can provide information through workshops with community-based organizations or brochures from state health agencies, such as MassHealth’s Redetermination Outreach Toolkit which provides a guide to approaching redetermination for patients [10]. In addition, health systems can engage volunteers or patient navigators to guide enrollees through the process, helping ensure that all required forms are filled out correctly. Recent health system initiatives provide templates for successful implementation, perhaps most notably with Temple Health’s financial counseling team, which has partnered with the Pennsylvania Department of Human Services to distribute informational flyers and posters throughout physician offices. Temple Health has also developed letters with details about the renewal process and contact information for county offices and change centers, with such instructions given to patients upon arrival to the clinic but also uploaded via MyChart for greater accessibility [11]. Although these options may increase administrative burden, stabilizing coverage is beneficial for health systems as well as patients, preventing hospital strain and offsetting costs from more complex disease presentation [12]. By actively engaging their patients with outreach efforts, health systems can uniquely protect patients’ access to Medicaid, particularly among minorities and non-native English speakers. Marginalized patients have already been slipping through the cracks, but this is preventable.

Beyond supporting patients through Medicaid redetermination, physicians should research and advocate for more permanent policy solutions [13]. Through revealing massive health inequities, the pandemic laid bare the need for physician advocacy. The past decade has seen an increase in the number of professional organizations with policy-focused committees and health policy mentorship to increase the civic engagement of clinicians. Indeed, voicing concerns at state and county health meetings, organizing and leading coalitions of non-profit organizations dedicated to increasing health access, and engaging with the media are all examples of how physicians can influence change. For example, after the untreated water in the Flint River resulted in elevated chloride levels, Dr. Hanna-Attisha’s research revealed that the number of individuals with high lead levels more than doubled after the switch, particularly affecting the most disadvantaged neighborhoods. Following the study's findings, residents were warned against consuming tap water. While doctors need not be political, they can and should advocate for data-driven policy that makes their patients safer, particularly with the respect and status that they are given in many communities. Ultimately, the path to equity and improved health via coverage can be paved through physician advocacy.

It has taken a global catastrophe to shift our healthcare system to one that considers access, albeit via a set of temporary reforms. However, these programs are but a band aid solution and are now being revoked without recourse or a permanent solution. This will not only threaten the health of underserved communities but also risks entrenching their distrust in the healthcare system, in which access is as capriciously revoked as given [14]. Clinicians can and must bridge this gap, ensuring access to individuals who need it the most.

## Data Availability

Not applicable.
